# Quality of life among Syrian refugees in Germany: a cross-sectional pilot study

**DOI:** 10.1186/s13690-021-00745-7

**Published:** 2021-11-29

**Authors:** Feras Al Masri, Mattea Müller, Josefine Nebl, Theresa Greupner, Andreas Hahn, Dorothee Straka

**Affiliations:** 1 Gottfried Wilhelm Leibniz University of Hannover, Institute of Food Science and Human Nutrition, Hannover, Germany; 2 Osnabrück University of Applied Sciences, Agricultural Sciences and Landscape Architecture, Osnabrück, Germany

**Keywords:** Quality of life, Syrian refugees, Physical health, Psychological, Social relationship

## Abstract

**Background:**

More than 10 million Syrians have left their homes and sought refuge in neighboring countries, including Europe, since the beginning of the Syrian conflict in March 2011, and immigration continues to this day. This cross-sectional study included Syrian refugees residing in and around Hannover, Germany. We investigated whether general socioeconomic factors (e.g. age, sex, housing, asylum duration) were predictive factors for the quality of life (QOL) of Syrian refugees in Germany.

**Methods:**

The QOL of Syrian refugees was assessed using the WHOQOL-BREF tool, a questionnaire assessing the QOL in four domains: Physical health, psychological, social relationships and environment. A total of 114 Syrian refugees, aged between 18 and 45 years, who obtained one of the following statuses, asylum, refugee protection or subsidiary protection, were included. The QOL domain and total scores of Syrian refugees in Germany were compared with a Western norm and Sub-Saharan population. Data were analyzed with the Spearman Rho correlation coefficient, Kruskal–Wallis and Mann–Whitney U test and multivariate linear regression.

**Results:**

More than 65% of the participants (62.3% male, 37.7% female) were between 18 and 29 years old, and 45% had lived in Germany for less than four years. The lowest QOL score was reported in the social relationship’s domain (60.5%), while the psychological score was lowest in participants aged 40–45 years (*P* = 0.011). The age was significantly negatively associated with physical health (*P* = 0.010), psychological (*P* <  0.001) and the total QOL (*P* = 0.005). Asylum duration was associated with the environment domain (*P* = 0.040), the short-time refugees were less satisfied than the longtime refugees, and with aspects of the psychological domain in Enjoying life and Concentration ability (*P* <  0.001 and *P* = 0.033, respectively), yet was not associated with total QOL or total domain scores. There were significant associations between housing and the psychological domain (*P* = 0.032) and housing and the social relationship domain (*P* <  0.001). The refugees who living in camps registered a lower score in psychological than residents of apartments and houses, and the single refugees had a higher score than those married and divorced (*P* = 0.032 and *P* = 0.035, respectively).

**Conclusions:**

The Syrian refugees participating in this study showed a low QOL score in the assessment of all domains compared to the normal population, especially regarding social relations and psychological; it was associated with socioeconomic factors, such as housing, asylum duration and marital status. This calls for urgent societal and political efforts to strengthen the social living conditions of Syrian refugees in Germany.

## Background

Ten years of violence have left a trail of destruction in Syria. Hundreds of thousands have died, millions have been displaced [1]. More than 10 million Syrians have left their homes and sought refuge in neighboring and European countries since the beginning of the Syrian conflict in March 2011 and immigration continues to this day [2]. As is well-known, wars and political conflicts lead to direct or indirect painfully experienced confrontations, such as seeing extreme violence, terrorist attacks, torture and kidnappings, which may lead to the dispersal of the family and the forced displacement of their members. Previous studies have shown that the local population of any region suffering from wars and political crises display symptoms of psychological shock or experience physical harm [3–5]. During and after the immigration process, refugees are, thus, faced with many challenges, such as previously suffered trauma as well as very different cultural and social environments in the country of asylum that influence their nutrition, health and quality of life (QOL) status [2, 6, 7]. Trauma is one putative factor in the development of diseases in refugee communities. In fact, studies suggest a positive association between trauma exposure and poor health in new refugees and asylum seekers [8–10]. Furthermore, the psychological state may affect the physical health and particularly the development of chronic diseases. It has been shown that traumatized Cambodian and Southeast Asian adults in the United States developed higher rates of diabetes and cardiovascular disease compared to the general population [10–12]. In other words, post-traumatic stress disorders, adaptation difficulties, loss of culture and the migration itself are closely related to the physical and psychological health of refugees [13, 14].

In Europe, Germany is one of the countries hosting the most international migrants and between 2015 and 2019 alone more than 1,622,954 people registered as asylum seekers with Syrians being the largest single nationality [15–19]. Within Germany, Syrian refugees were distributed differently in the German states: Most refugees were registered in North Rhine-Westphalia (24.3%), followed by Bavaria (13.1%) and Lower Saxony (9.60%) [20]. When comparing the Syrian refugee population from the migration wave of 2015 to the general German population, the Syrian refugees are, on average, younger and relatively well educated, with 25% of them having a high school diploma and higher levels of university or secondary education [20]. Moreover, more than 25% have worked in medical, technical, teaching and engineering professions in Germany [21]. Nevertheless, the percentage of wage-employed refugees among the Syrian refugees is low when compared to the local community, and their sources of income differ from the general population. Only 13% indicate that wage from work is their main source of income, and the main sources of income for more than 55% of refugees are unemployment benefits and other government support [22].

It has been generally noted that a higher perceived ethnic discrimination is associated with lower mental and physical health, but not for Syrian refugees, who have derived a sense of control, distinctiveness and meaningfulness from their Syrian identity. Ethnic pride is a protective factor that mitigates the effects of discrimination on the symptoms of depression and social bonding [23–25].

This large migration has triggered the focus of the current political agenda on factors important for the successful integration and QOL of the refugee population in Germany. Policies are called for to develop the economic measures of societal conditions and promote and increase the QOL of their citizenry directly, citizens and immigrants alike [26]. The concept of QOL includes a wide range of components defined by the Organization for Economic Co-operation and Development’s “How’s Life? Report,” including income, housing, health status, work, life balance, education, skills, social connections, civic engagement, environmental quality and personal security [27, 28]. However, data regarding the QOL of Syrian refugees residing in Germany since 2015 is scarce, and are only available on subjective questions on the general health status or satisfaction with the health system, which make it difficult to compare and evaluate the QOL status of Syrian refugees in Germany regarding the study.

Therefore, the present cross-sectional study focused on assessing the QOL using the well-established WHOQOL-BREF tool to evaluate the physical health, psychological health, social relationships and environment of Syrian refugees residing in Germany since 2015 who obtained one of the following statuses: Entitlement to asylum, refugee protection or subsidiary protection. Furthermore, we investigated whether general socioeconomic factors (e.g. age, sex, housing, asylum duration) were predictive factors for the QOL of Syrian refugees in Germany.

## Methods

### Study design and participants

**Fig. 1:**
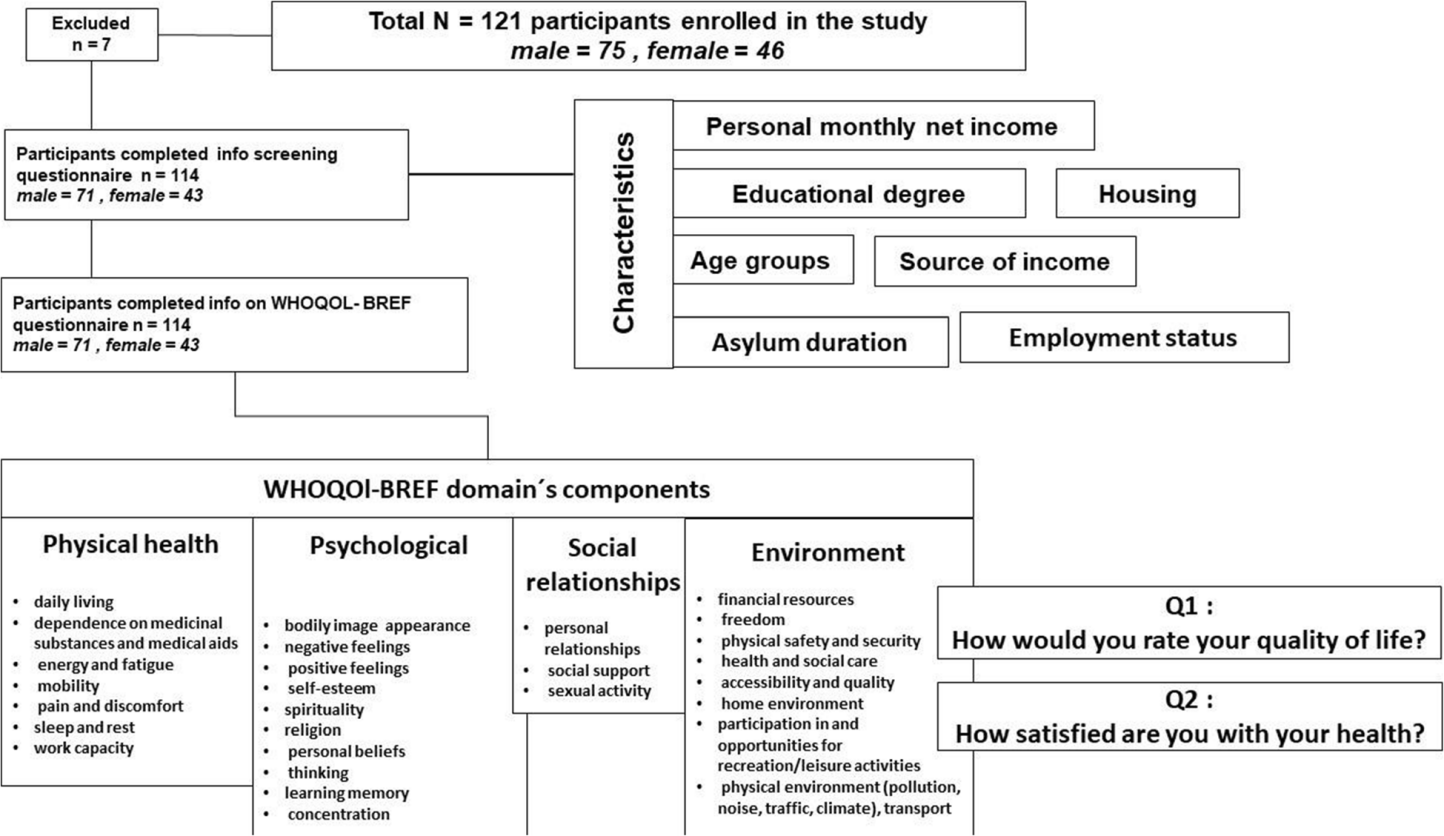
Study participant characteristics and method components of Syrian refugees in Germany 2018–2020

There are 15,485 foreigners of Syrian nationality (naturalized, immigrants, refugees and asylum seekers) residing in the city of Hannover [20]. This cross-sectional pilot study was conducted at the Institute of Food Science and Human Nutrition, Leibniz University Hannover, Germany, between December 2018 and Mars 2020 and included 114 Syrian refugees (Fig. 1). The study received ethical approval from the Ethics Committee at the University of Applied Sciences Osnabrück. All participants gave their written informed consent. The assessment and processing of the data were completed following the Lower Saxony Data Protection Act, adhering to the Declaration of Helsinki and the principles of Good Clinical Practice. The participants were recruited by announcing the study on social media, in the local press, refuge reception centers and language schools that conduct the integration courses. The participants were selected only from Syrian refugees who obtained one of the following statuses: asylum, refugee protection or subsidiary protection, and other residency types were excluded. The sample was selected to be representative regarding the sex and age distribution of the total refugee population that immigrated between 2015 and 2019, as reported by the Federal Office for Migration and Asylum in Germany (BAMF) [15–19]. Furthermore, inclusion criteria were age between 18 and 45 years, residing in and around Hannover, Germany, and asylum since 2015. The following criteria led to exclusion: Cardiovascular, metabolic or malignant disease, gastrointestinal diseases, pregnancy, food intolerances and addiction to drugs or alcohol. The participants were invited to the university to complete the WHOQOL-BREF questionnaire regarding QOL and the latter was assessed individually during a face-to-face interview with each participant. A screening questionnaire assessing sociodemographic characteristics was filled in by the participants before the interview. The number of interviews reached 114 in 55 study days; the number of participants in each study day ranged from one to a maximum of six (Fig. 1).

### Quality of life questionnaire

**Fig. 2:**
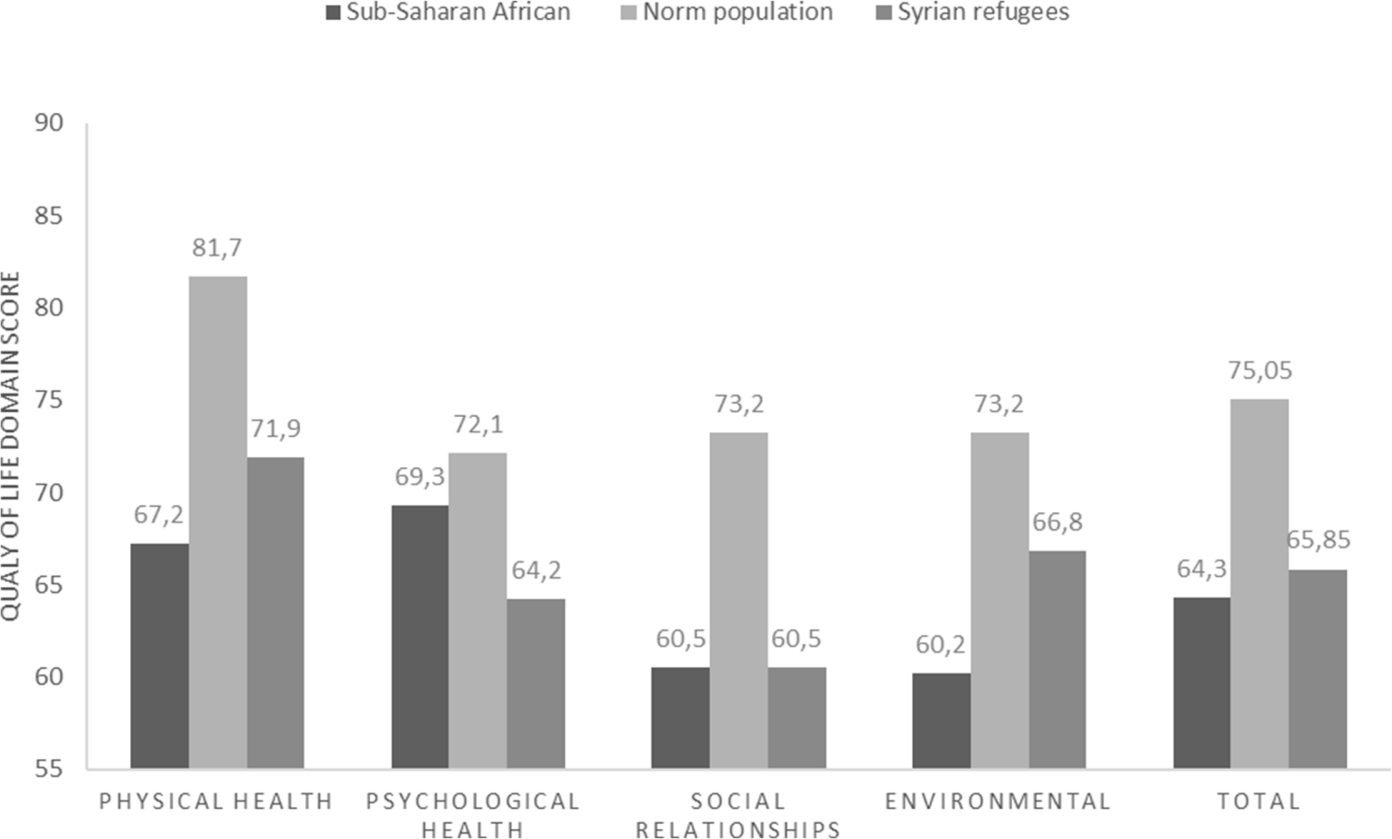
Quality of life of Syrian refugees in Germany in 2018–2020 comparison with West norm population [1] and sub-Saharan African migrants in Germany [3] according to (World Health Organization quality of life Instrument, Short Form)

The World Health Organization (WHO) defined QOL as “an individual’s perception of their position in life, in the context of the culture and value systems in which they live, and concerning their goals, expectations, standards, and concerns.” [29] The WHOQOL-BREF is a validated questionnaire assessing the QOL in four domains: Physical health, psychological social relationships and environment [30–32]. The WHOQOL-BREF contains of 26 questions with structured responses with Likert-style response scales: Very poor to very good (evaluation scale), very dissatisfied to very satisfied (evaluation scale), none to extremely (intensity scale), none to complete (capacity scale) and never to always (frequency scale) [35]. Each domain consists of questions for which the scores vary between one and five. The mean score in each domain indicates the individual’s perception of their satisfaction with each aspect of their life, relating it to the QOL [32]. Two of the 26 questions, questions 1 and 2 (Q1, Q2), assess the QOL and health in general as perceived by the participant, and the others (24 questions) comprise specific details of the four domains: Physical health, psychological, social relationships and environment (Fig. 1). The questionnaires were completed during face-to-face interviews; the questions were translated from the English version to the Arabic language orally by one researcher speaking both Arabic and English and using Arabic terms that are consistent with the general vision of the question. As the sample included Arab and non-Arab Syrian ethnicities, such as Kurdish, Assyrian, and Syriac, who speak Arabic, but are unable to read or write it properly, the English version was chosen after being matched with the Arabic version, to ensure that there is no bias towards the Arab-Syrian participants. This method has been used to facilitate the understanding of the questions and clarify them to the participants in a way that is appropriate to their mother tongue, while explaining the answer mechanism and its purpose without affecting the personal and self-evaluation of the answers. In order to put these data into context of previous studies, a descriptive comparison of the total and domain QOL scores was made with either a Western norm population [33] and Sub-Saharan African immigrants in Germany by Adedeji [34] as a basis comparison of QOL scores (Fig. 2).

### Statistical analysis

The descriptive of the study population included percentages of sociodemographic and economic characteristics, means of QOL domain scores and standard deviations, Medians of domains questions and (25–75% percentile). The WHOQOL-BREF questions are answered with a 5-point Likert scale and range from 1 (very poor/very dissatisfied/none/never) to 5 (very good/very satisfied/very/always), then scores for all four domains are summed and converted into a percentage, with higher scores indicating better quality [35, 36]. The reliability the WHOQOL-BREF questionnaire showed good internal consistency (Cronbach’s alpha of 0.876), which is in line with previous studies [37–42].

Spearman Rho correlation coefficient was used to determine the level of agreement between the four domains of the WHOQOL-BREF, the four domains with each other and age groups, duration of asylum, educational degree and monthly net income. The Spearman Rho correlation coefficient was also used to determine the level of agreement between asylum duration and each component in the tool.

A Kruskal–Wallis, Mann–Whitney U test and linear regression design were used to compare the WHOQOL-BREF and characteristics (sex, age groups, duration of asylum in Germany, professional degree and employment status) to determine associations between sociodemographic characteristics and WHOQOL-BREF. The non-normally distributed data were transformed using the square root function before multiple linear regression. If there were significant differences between the groups, the post hoc test with least significance difference correction was performed. All statistical analyses were conducted using SPSS software (IBM SPSS Statistics 26.0.0.0; Chicago, IL, USA). The scores were used for statistical analyses for all domains and the level of significance was set at *P* <  0.05.

## Results

### Participant characteristics

In this study, 62.3% (*n* = 114) of the participants were male and 37.7% were female (Table 1). About 45% of the Syrian refugees had lived in Germany for less than four years; 30% between one and three years. Notably, about half of the sample had a university degree and more than 33% had completed high school. Furthermore, more than 85% lived on less than 1000 € income per month and only 13% had between 1000 and 2000 € at their disposal from various sources, such as unemployment benefits or work. Furthermore, 78% of participants lived in rented apartments and less than 3% lived in refugee camps. It is worth noting that these data are comparable to those reported by the Federal Office for Migration and Asylum in Germany (BAMF), where the 64.3% were male and 35.7% women, the university graduates exceeded 30%, and the percentage of the age group between 18 to 30 years amounted to 69% [15–19].

**Table 1:** Characteristics of the study Syrian Refugees in Germany in 2018–2020 (*N* = 114)

Sociodemographic and economic characteristics n (%)			
**Sex**	**Duration of asylum**	**Housing**	**Employment status**
Male: 71 (62.3)	< 1 year: 27 (23.7)	Rented house: 22 (19.3)	Employed in Syria and Germany: 25 (21.9)
Female: 43 (37.7)	1 year - > 3 year: 32 (28.1)	Rented apartment: 89 (78.1)	Employed in Syria, unemployed in Germany: 41 (36.0)
Total: 114 (100)	> 3 year: 55 (48.2)	Refugee camp: 3 (2.60)	Unemployed in Syria, employed in Germany: 18 (15.8)
**Age groups (years)**	**Educational degree**	**Marital status**	Unemployed in Syria and Germany: 30 (26.3)
18–24: 35 (30.7)	No education: 16 (14.0)	Single: 80 (70.2)	**Personal monthly net income**
25–29: 40 (35.1)	Basic education: 7 (6.10)	Married: 29 (25.4)	< 1000 €: 97 (85.1)
30–34: 21 (18.4)	Secondary: 38 (33.3)	Divorced: 5 (4.4)	1000 – < 2000 €: 15 (13.2)
35–39: 11 (9.60)	University: 51 (44.7)		2000–3000 €: 2 (1.80)
40–45: 7 (6.10)	Postgraduate: 2 (1.80)		

### Quality of life scores

The results of QOL as measured by the WHOQOL-BREF are presented as a QOL score in four domains: physical health, psychological health, social relationships and environmental health.

As in Fig. 2 showed, the QOL scores of Syrian refugees were lower than West norm population scores in All domains and total scores, while there were higher than Sub-Saharan African in Physical health, environmental, and total. In contrast the psychological health was higher in Sub-Saharan than Syrian refugees and similar in social relationships domain.

The total QOL score and the scores of the four domains: physical health, psychological, social relationships and environment, are presented in Table 2. The total QOL score was an average of 65.9 ± 12.7. Among the domains, the physical health domain recorded the highest mean score of 71.9 and a minimum score of 31.0. The psychological domain reported a mean score of 64.2 ± 16.5 and a minimum score of 19.0. The social relationship domain had the lowest mean score of 60.5 ± 20.3 and a minimum score of 6.00. Finally, the environment domain recorded a mean score of 65.9 ± 12.7 and a minimum score of 25.0.

**Table 2:** Comparison of the quality of life scores (World Health Organization quality of life Instrument, Short Form) according to independent variables of Syrian refugees in Germany in 2018–2020

Characteristics	Physical health (QOL score) ± SD	Psychological (QOL score) ± SD	Social relationships (QOL score) ± SD	Environment (QOL score) ± SD	Total (QOL score) ± SD
**Total (n)**	71.9 ± 13.8	64.2 ± 16.5	60.5 ± 20.3	66.8 ± 14.1	65.9 ± 12.7
**Sex**					
Male	73.0 ± 14.0	66.0 ± 17.0	60.0 ± 21.0	68.0 ± 15.0	67.0 ± 13.4
Female	70.0 ± 16.0	61.0 ± 14	61.0 ± 19.0	65.0 ± 13.0±	64.0 ± 112
**P Mann-Whitney -U-**	0.165	0.044*	0.876	0.209	0.235
**Spearman Rho r**	−0.131	− 0.190**	0.15	− 0118	−0.112
**Education degree**					
No education	67.0 ± 17.0	59.0 ± 21.0	65.0 ± 20.0	63.0 ± 16.0	63.5 ± 18.5
Basic education	71.0 ± 17.0	57.0 ± 19.0	52.0 ± 16.0	68.0 ± 13.0	62.0 ± 16.3
Secondary	67.0 ± 9.00	67.0 ± 9.00	62.0 ± 15.0	70.0 ± 15.0	68.8 ± 12.0
University	72.0 ± 13.0	65.0 ± 17.0	60.0 ± 21.0	67.0 ± 14.0	66.0 ± 16.3
Postgraduate	69.0 ± 8.00	50.0 ± 27.0	50.0 ± 27.0	69.0 ± 8.00	59.5 ± 17.5
**P Kruskal-Wallis**	0.767	0.527	0.773	0.750	0.707
**Spearman Rho r**	0.100	0.149	0.031	0.077	0.106
**Age groups (years)**					
18–24 (35)	72.0 ± 13.0	65.0 ± 14.0	63.0 ± 19.0	69.0 ± 14.0	67.2 ± 12.2
25–29 (40)	75.0 ± 14.0	69.0 ± 15.0	62.0 ± 22.0	67.0 ± 15.0	68.5 ± 12.3
30–34 (21)	70.0 ± 12.0	60.0 ± 19.0	58.0 ± 23.0	66.0 ± 12.0	63.5 ± 13.5
35–39 (11	72.0 ± 14.0	59.0 ± 19.0	57.0 ± 17.0	63.0 ± 17.0	62.7 ± 11.6
40–45 (7)	58.0 ± 16.0	49.0 ± 14.0^a^	55.0 ± 18.0	63.0 ± 13.0	56.3 ± 13.5
**P Kruskal-Wallis**	0.086	0.011*	0.812	0.636	0.180
**Spearman Rho r**	−0.103	− 0.195**	− 0.109	−0.131	− 0.149
**Housing**					
Rented house (22)	74.0 ± 12.0	67.0 ± 11.0	63.0 ± 17.0	66.0 ± 14.0	67.5 ± 8.8
Rented apartment (89)	72.0 ± 14.0	64.0 ± 17.0	61.0 ± 20.0	67.0 ± 14.0	66.1 ± 13.2
Refugee camp (3)	62.0 ± 16.0	46.0 ± 13.0	23.0 ± 19.0 ^b^	57.0 ± 11.0	46.9 ± 7.1
**P** ***Kruskal-Wallis***	0.624	0.113	0.030*	0.435	0.106
**Marital status**					
Single (80)	72.0 ± 14.0	67.0 ± 15.0	62.0 ± 19.0	67.0 ± 14.0	67.1 ± 12.5
Married (29)	72.0 ± 14.0	59.0 ± 18.0^c^	58.0 ± 22.0	67.0 ± 15.0	63.8 ± 13.5
Divorced (5)	66.0 ± 13.0	44.0 ± 14.0^c^	41.0 ± 28.0	69.0 ± 8.00	54.69 ± 10.1
**P** ***Kruskal-Wallis***	0.730	0.033*	0.444	0.997	0.292
**Asylum Duration**					
< 1 year (27)	74.0 ± 15.0	69.0 ± 16.0	64.0 ± 20.0	67.0 ± 14.0	68.5 ± 14.7
1 year - > 3 year (32)	73.0 ± 14.0	64.0 ± 15.0	59.0 ± 21.0	71.0 ± 14.0	76.7 ± 12.5
> 3 year (55)	70.0 ± 13.0	62.0 ± 17.0	60.0 ± 20.0	65.0 ± 14.0	64.1 ± 12.0
**P** ***Kruskal-Wallis***	0.562	0.210	0.540	0.086	0.442
**Spearman Rho r**	0.288	0.108	0.324	0.277	0.206

QOL score: Mean of QOL in percentage, *P <  0.05, ** Spearman Rho r <  0.05, ^a^ significant post hoc test (*P* <  0.05) comparing age group 40–45 years to all age groups in the psychological domain, ^b^ significant post hoc test (*P* <  0.05) comparing refugees living in refugee camps with all groups in the social relationships’ domain, ^c^ significant post hoc test (*P* <  0.05) comparing single to married, single to divorced, total *N* = 114

### Differences in QOL scores according to sociodemographic characteristics

There were no significant differences in the four domains of QOL between sex, asylum duration and education except the psychological domain in sex (Table 2). However, in terms of absolute changes, men had slightly higher QOL scores compared to women. Refugees who were in the first year of residency in Germany scored slightly higher in all domains, except for the environment domain.

Interestingly, when comparing the QOL scores between age groups, the psychological score was significantly lower in 40–45-year-old refugees compared to the younger age groups. Moreover, a significant inverse correlation was found between psychological and age groups (*P* = 0.011). In addition, a significance statistic appeared in the psychological domain regarding marital status (*P* = 0.006). The refuges who were single had a higher psychological score compared to those who were married or divorced; in addition, the married participants had significantly higher psychological scores than those divorced (Table 2).

Furthermore, the total QOL scores and the scores in the social relationship domain were significantly lower among participants who live in refugee camps compared to other housing (*P* = 0.028), while there were no significant differences between the other characteristics (Table 2). However, is should be noted that only three participants lived in refugee camps at the time of the study.

The results from the multiple linear regression, as presented in Table 3, show a significantly negative association between age and physical health (*P* = 0.02) and psychological domain (*P* = 0.007). Further significant associations were observed between housing with the psychological and relationship domains. However, the refugees who were living in camps (only three participants) registered lower scores in the psychological domain than residents of apartments and houses (*P* = 0.018). In addition, the environment domain was negatively associated with the age (*P* = 0.033). The total QOL score was negatively associated with age (*P* = 0.005) and housing (*P* = 0.008). The were no significant differences between characteristics such as sex, educational degree, personal monthly net income and source of income.

**Table 3:** Multiple linear regression analyses of significant factors associated with the quality of life (World Health Organization quality of life Instrument, Short Form) of Syrian refugees in Germany in 2018–2020

QOL domains	Factors associated with the domain	Standardized coefficients		***P***-value	Confidence intervals	
		Beta	T		Under	Upper
**Physical health**	Age	−0.35	−3.15	0.002*	− 0,08	− 0,02
**Psychological**	Age	−0.30	−2.74	0.007*	− 0.09	− 0.02
	Housing	−0.21	−2.40	0.018*	− 2.48	− 0.69
**Social relationships**	Housing	−0.38	−4.24	< 0.001*	−5.05	−4.76
**Environment**	Age	−0.25	−2.17	0.033*	−0.07	− 0.004
**Total**	Age	−0.30	−2.71	0.005*	−0.07	− 0.01
	Housing	−0.25	−2.75	0.008*	−2.13	−0.35

*P value < 0.05*,* total *N* = 114

### Differences in the QOL domains’ components score according to the asylum duration

Although asylum duration was not directly associated with the total QOL scores and scores of the four domains, correlation analysis showed significant associations to asylum duration exclusively in the psychological domain.

Considering the duration of asylum specifically, the WHOQOL-BREF revealed that group 1 (< 1 year asylum duration) was higher than the other groups in the axis of enjoying life, concentration ability, money availability, information availability and transportation. On the other hand, the healthcare access satisfaction component was higher in group 3 (> 3 years duration) than in the other groups (Table 4).

**Table 4:** Quality of life (World Health Organization quality of life Instrument, Short Form) version domains components of Syrian refugees in Germany in 2018–2020 according to asylum duration

Domains’ components	Group 1 < 1 year ***n*** = 27	Group2 1–3 years ***n*** = 32	Group3 > 3 years ***n*** = 55		
	Median (25–75% percentile)	Median (25–75% percentile)	Median (25–75% percentile)	*P*-value	Spearman Rho ***r***^***1***^
**Psychological domain**					
f5: Enjoying life	4.00 (3.00–4.00)	3.00 (3.00–4.00)	3.00 (3.00–4.00)	< 0.001*	−0.323**
f7: Concentration ability	4.00 (3.00–4.00)	4.00 (3.00–4.00)	3.00 (3.00–4.00)	0.033*	−0.226**
**Environment domain**					
f12: Money availability	4.00 (3.00–4.00)	4.00 (3.50–5.00)	4.00 (3.00–4.00)	0.042*	−0.061
f13: Information availability	4.00 (3.00–4.00)	4.00 (3.00–5.00)	4.00 (3.00–4.00)	0.026*	−0.234**
f24: Healthcare access satisfaction	3.00 (2.00–4.00)	4.00 (3.00–3.00)	4.00 (4.00–4.00)	0.023*	0.219**
f25: Transportation	4.00(4.00–4.00)	4.00(3.00–4.00)	4.00(3.00–4.00)	0.043*	− 0.206*

*p-value < 0.05, ** Significant Spearman Rho r, ^1^ Spearman Rho *r* correlation between QOL-BREF questions and asylum duration, Total *N* = 114, NB: A score of 5 on the Likert scale means very satisfied or very good, and 1 means very dissatisfied or very poor [43]

By contrast, considering subjective questions, the physical health and social relationship domain, there were no differences between the three groups. It should be noted that the components which were not significant were removed from Table 4.

### General view of QOL and health

Table 5 depicts the subjective assessment of the general QOL and general health and the corresponding QOL mean scores. The means were significantly different between all WHOQOL-BREF domains in the subjective general QOL question: “How would you rate your quality of life?” (Q1): Physical health (*P* < 0.005), psychological (*P* < 0.001), social relationship (*P* = 0.016), environment (*P* = 0.007) and the total score (*P* < 0.001). They were also significantly different between all domains in the general health question: “How satisfied are you with your health?” (Q2): Physical health (*P* < 0.001), psychological (*P* < 0.001), social relationship (*P* = 0.032), environment (*P* = 0.003) and the total score (*P* < 0.001). These significant differences appeared in the least satisfied groups (very poor, poor and neither poor nor good), where a significantly lower QOL score was recorded compared to the most satisfied groups, although the least satisfied groups accounted for 12.20% in (Q1) and 18.40% in (Q2) of the total sample.

**Table 5:** Comparison of the quality of life (World Health Organization quality of life Instrument, Short Form) domain with question 1 and question 2 of Syrian refugees in Germany in 2018–2020

**General QOL (Q1)**	**n (%)**	**Physical health**	**Psychological**	**Social relationships**	**Environment**	**Total**
		QOL score ± SD	QOL score ± SD	QOL score ± SD	QOL score ± SD	QOL score *±* SD
**Very poor**	3 (2.60)	48.0 ± 14.0^a^	34.0 ± 13.0 ^a^	40.0 ± 10.0 ^a^	52.0 ± 16.0 ^a^	49.5 ± 19.5 ^a^
**Poor**	11 (9.60)	61.0 ± 13.0 ^a^	42.0 ± 16.0 ^a^	53.0 ± 20.0 ^a^	62.0 ± 20.0 ^a^	54.5 ± 12.1 ^a^
**Neither poor nor good**	44 (38.6)	71.0 ± 13.0	61.0 ± 12.0 ^a^	58.0 ± 20.0 ^a^	64.0 ± 13.0 ^a^	63.4 ± 11.3 ^a^
**Good**	47 (41.2)	75.0 ± 12.0	71.0 ± 12.0	61.0 ± 18.0	69.0 ± 12.0 ^a^	69.1 ± 10.1 ^a^
**Very good**	9 (7.90)	80.0 ± 14.0	79.0 ± 17.0	81.0 ± 21.0	80.0 ± 15.0	79.9 ± 11.4
**P Kruskal-Wallis**		0.005*	< 0.001*	0.016*	0.007*	< 0.001*
**General Health (Q2)**	**n (%)**	**Physical health**	**Psychological**	**Social relationships**	**Environment**	**Total**
		QOL score ± SD	QOL score ± SD	QOL score ± SD	QOL score ± SD	QOL score ± SD
**Very dissatisfied**	5 (4.40)	50.0 ± 17.0 ^a^	41.0 ± 14.0 ^a^	46.0 ± 14.0 ^a^	59.0 ± 24.0 ^a^	49.2 ± 15.0 ^a^
**Dissatisfied**	16(14.0)	65.0 ± 11.0 ^a^	53.0 ± 16.0 ^a^	51.0 ± 23.0 ^a^	58.0 ± 11.0 ^a^	56.8 ± 11.4 ^a^
**Neither satisfied nor dissatisfied**	40 (35.1)	70.0 ± 13.0 ^a^	62.0 ± 16.0 ^a^	61.0 ± 19.0	65.0 ± 15.0 ^a^	64.4 ± 11.5 ^a^
**Satisfied**	43 (37.7)	76.0 ± 11.0	70.0 ± 13.0	64.0 ± 19.0	69.0 ± 11.0	69.8 ± 9.80
**Very satisfied**	10 (8.80)	83.0 ± 10.0	79.0 ± 13.0	70.0 ± 24.0	79.0 ± 12.0	77.7 ± 11.9
**P Kruskal-Wallis**		< 0.001*	< 0.001*	0.032*	0.003*	< 0.001*

QOL score: Mean of QOL in percentage. *P < 0.05. N total = 114. ^a^ significant post hoc test (*P* < 0.05)

## Discussion

This study highlighted the QOL of Syrian refugees in Germany through the four main domains and their respective components and predicted the socio-demographic factors that could be associated with QOL. This may lead to some extent to the prediction of new possibilities for intervention, by focusing more attention on the most influential components of QOL domain such as enjoying life and concentration ability in psychological domain and money availability, information availability, healthcare access satisfaction, and transportation in environment domain.

However, extending this pilot study to a full-fledged study may be useful for governments in addressing the poor QOL and thus the level of refugee integration and effectiveness in host communities. Furthermore, in order to move from this pilot study towards a full study, based on the observed progression of the study methodology, the number of interviews per day should be increased by increasing the number of trained interviewers. Taking into consideration the ethnic diversity of the Syrians and the consequent difference in culture and language between the ethnicities of Syrians, whether Arab, Kurdish, or other ethnic minorities. In addition, the different of the area’s nature from which refugees originate, whether urban or rural, which were not discussed in this pilot study may contribute in some way to deepening the understanding of the role of socio-demographic factors in affecting the QOL. Moreover, limiting the current study to refugees who were granted asylum or subsidiary protection only and excluding asylum seekers somehow missed the opportunity to know the extent and speed of change in the QOL scores.

There is currently a lack of knowledge about the QOL of Syrian refugees in Germany as well as the potential predictive factors between the QOL domains (physical health, psychological, social relationships, and environment) and sociodemographic characteristics. Therefore, this cross-sectional study investigated the QOL and potential factors affecting QOL (i.e. sociodemographic characteristics, duration of asylum, work status, marital status, monthly net income and housing status) of Syrian refugees who have migrated to Europe since 2015.

About 46.5% of the refugees hold high educational and professional qualifications. Most of them are engaged in social activities in Germany, such as language schools, integration courses, schools and universities, and some are working in official jobs after they had been legally registered as refugees. However, most of them still depend on government aid and subsidies as a source of income with a personal monthly net income of less than 1000 €, which is consistent with reports from the German Federal Office for Immigration and Asylum [22, 44].

### Total QOL score

This study reported a total QOL mean score of 65.9 ± 12.7% regarding Syrian refugees in Germany, which is lower compared to the normal population (75.5%) [33]. Nevertheless, the Syrian participants in this study scored higher levels of QOL in all domains when compared to other refugee populations, for example, sub-Saharan African migrants in Germany with a total QOL mean score of 64.3% [33, 34]. The poor QOL score indicates that physical health and social needs are not fully met by Syrian refugees. This may be partly due to racial discrimination and feelings of hostility which may affect the QOL of refugees in German society, as reported by studies in other societies [45, 46].

The study also showed a significant correlation between the subjective assessment of QOL (Q1) and health (Q2), on the one hand, with objective assessments of the four QOL domains, on the other hand. However, this correlation appeared in the worst conditions and at the lowest levels of satisfaction. Which was consistent with other studies reporting on the relationship between objective evaluation and the subjective assessment of QOL [47, 48]. This may be explained by the effects of post-traumatic stress disorders and the difficulty of overcoming their effects with the increasing pressure of adapting to the new country [3].

### Physical health

In this study, the physical health domain has the highest score compared to the other three domains, which is also compatible with previous Jordanian, Iraqi and Swedish studies on Syrian refugees, in addition to the Dutch study on Somali and Iranian refugees [6, 7, 9, 49]. This is probably related to the young age of the study population with most of them in good physical health status [15, 44].

The physical health domain score was 71.9%, which was higher compared to two other studies carried out on Syrian refugees in Jordan (48.6%) and Syrian refugees in refugee camps in Iraqi Kurdistan (66.3%). Both studies used the WHOQOL-BREF to investigate the QOL of adult male and female refugees with study populations of *n* = 270 in Iraq and *n* = 655 in Jordan [6, 7]. This difference may be largely due to the asylum conditions and the fact that Syria’s neighboring countries, such as Jordan and Iraq, are the first direct destination for Syrian refugees from the conflict zone to a more stable region. This situation coincided with elements that negatively affected physical health, such as crowding, sadness, loss of dignity, guilt, despair, lack of confidence and shame. While Europe was the second resort after the refugees had improved their relative stability in the health and psychological statuses [50].

The Syrian refugees in this study reported higher levels of satisfaction with their physical health compared to the sub-Saharan African migrant population in Germany using the same tool to assess QOL [34]. This difference might be due to the different duration of asylum between Syrians and sub-Saharan Africans since the sub-Saharan African immigrants have already resided in Germany for up to ten years compared to the maximum of four years for the Syrian refuges in this study [51]. The physical health of longtime refugees was lower compared to the short-time refugees, as observed in a Dutch study of Iraqi refugees in the Netherlands which compared two groups of refugees according to their asylum duration (less than six months and more than two years) and a Norwegian study using the WHOQOL-BREF tool for Syrian refugees coming from Lebanon. The studies attributed this to the fact that the long asylum procedures may play a negative role in affecting the physical health and QOL of refugees [51, 52]. The QOL in another Swedish study with short-time asylum duration refugees utilized the WHOQOL-BREF tool twice: The first time after six months and the second after one year of asylum. The study indicated that the physical health of the longtime refugees was lower compared to that of the short-time refugees, this could be explained by the influence of post-traumatic stress disorder on their adaptation to cultural changes [53]. However, in this study, we did not find a significant correlation between the asylum duration and the physical health of the participants, which might be due to the more favorable asylum conditions. Nevertheless, the immigrants from both the sub-Saharan African migrants and the present study reported lower levels of satisfaction with their physical health compared to a norm population [33].

Finally, it can be said that the main determinant related to physical health is age, consistent with a German study reporting on about 7000 refugees of many nationalities including Syrians and other studies in Europe [39, 54, 55].

### Psychological domain

The mean score of the psychological domain for participants was 64.2 ± 16.5%, which was higher compared to Syrian refugees in Jordan (53.8%) and in the refugee camps in Iraqi Kurdistan (49.8%) [6, 7]. However, on the other hand, the mean score of the psychological domain for the Syrian refugees was lower compared to the sub-Saharan African migrants (69.3%) and the norm population (70.6%).

In this study, the psychological domain of refugees was negatively correlated to age, which was consistent with other studies of Syrian refugees in Turkey [56] and The Netherlands, [9] and tortured refugees in Denmark [57, 58]. As noted previously, this study included only 8.7% of refugees in the highest age group between 40 and 45 years, however, this group showed clear dissatisfaction in the psychological domain. Nevertheless, this was not as pronounced compared to Afghan, Iranian and Somali refugees in a Dutch study, the Somali refugees in a USA study and Syrian refugees in a Turkish study [56, 59, 60]. This may be explained by the fact that younger participants experience lower anxiety and illness due to their ability to adapt to new environmental conditions better and faster [61].

Refugees living in refugee camps in this study had the lowest psychological health score (46%), although it should be noted this only involved three participants. However, this is a tendency which was observed in other studies showing the lowest psychological scores for those living in refugee camps compared to other housing. The reason for this may be the strict rules in the refugee camps and uncertainty regarding the living situation, leading to a higher rate of anxiety, depression and post-traumatic stress disorders [62–64]. The psychological domain score was not affected by marital status, which was in contrast to that was reported in a German study conducted on 119 Syrian refugees in Germany. It should be noted that data on the time and location of the of separation or divorce were not available [65, 66].

Newly arrived refugees generally reported moderate to good levels of QOL and a low prevalence of mental illness in an Australian study of 233 new immigrants from Africa, South Asia, the Middle East and West Asia [67]. The Dutch and Swedish studies also reported that the short-time refugees had better psychological domain scores when compared to the longtime refugees [51, 53]. This is not the case in the present study, where there were some negative associations when looking more closely at the single questions regarding the psychological domain in terms of enjoyment of life and the ability to concentrate. Perhaps this might explained by better asylum conditions, such as financial aid and health services [63].

The results also recorded a significant correlation between the psychological domain score and the components of physical health, social relationships and, to a lesser extent, the environment. These results are consistent with what has been observed in many previous studies on different groups of refugees and their host and countries [68–70].

### Social relationships

The social relationships scored lowest at 60.5% when compared to other domains. The satisfaction regarding social relationships in other studies with Syrian refugees was lower in Jordan (49.8%), while it was higher in Iraqi Kurdistan (70.4%) and the Gaza Strip (71.4%) compared to the Syrian refugees in this study [6, 7]. These results may be explained by the circumstances of the asylum, the same language, and the social religious and cultural background being more similar to the refugees’ own cultural origins.

Moreover, previous studies observed an important effect of gender differences on the QOL, especially in the social relationships domain, as the study of sub-Saharan African migrants reported a better degree of QOL for immigrant women. This is in line with the present study, as there was a slightly better score among Syrian refugee women,despite the absence of significance between men and women in the social relationships domain [34]. This contradicts other studies, as it was associated with males being affected by social relationships in a positive way more than females, [71] and this may be related to the social and religious heritage of Syrians, as males are responsible for fully supporting their families and they carry additional social and economic burdens that may be related their social environment [72].

It is noteworthy that social relationships show an association with housing in the present study with Syrian refugees in Hannover. Whereas the camp residents recorded a low score for social relationships compared to the houses and apartments residents. The reason for the low score regarding social relationships in this study may be the large cultural differences including social and religious habits in addition to the language and communication barrier [73].

### Environment domain

The mean score of QOL in the environment domain in this study was 66.8%. It was higher than that observed in the Iraqi (46.58%) and the Jordanian study (47.37%). This difference can be explained by the better environmental conditions in Germany as a high-income country, such as health services, transport and housing [6, 7, 57, 58]. Although a higher satisfaction is recorded among Syrian refugees compared to the sub-Saharan African refugees in Germany (60.2%), it was still lower when compared to the norm population (73.1%) [33, 34].

In this study, the short-time refugees were less satisfied than the longtime refugees in terms of healthcare access and feeling secure, which was consistent with a Swedish study [53]. However, the scores of the short-time refugees were higher than the longtime ones regarding the physical environment, money availability, information availability, opportunity for leisure activities, living place environment and transportation. This was also consistent with the Dutch study [51]; this difference may be explained by the different coping styles and personalities among individuals, and is due to a personal evaluation of the environment [74, 75].

The mean score of the environment domain was negatively correlated with age; this may be due to the higher adaptation skills among the younger refugees than the older ones. This was confirmed by previous studies, which showed a higher ability of the youngest to adapt to new conditions with a higher efficiency in Iraqi refugees in Malaysia [61]. In addition, the environmental domain was positively correlated with all components of other domains, especially the psychological domain, as reported by the European study on local population communities, which included the German host community [70].

### Limitations and strengths

A major limitation of this study is the cross-sectional design which limits causal interpretation. Additionally, the pilot study has a small sample size. The response rate was low, as many of the potential participants had political and social concerns despite the guarantee of anonymity of the participants. In addition, as individuals in the current sample were refugees who had obtained the right of asylum or subsidiary protection and although the sampling method was taken to represent the Syrian refugee population in Germany, we cannot exclude the possible selection bias, especially since the sample was recruited only from Hannover. Moreover, generalizing the results may be limited to refugees who had arrived in Germany and obtained asylum during the period of the Syrian crisis, and were not included with asylum seekers. However, all participants were carefully and individually instructed concerning the QOL questionnaire, which ensured full compliance without missing data. An attempt was made to reduce potential cultural barriers by explaining the scientific background of the questions, such as “How satisfied are you with your sexual life?,” in the mother language of the participants.

## Conclusion

The present study provides practical information for policy makers and public health officials about the QOL scores and influencing factors among Syrian refugees in Germany. The Syrian refugees participating in this study showed a low QOL score in the assessment of all domains, especially in the social relationships and psychological domains, when compared to the norm population. Sociodemographic factors, especially age, housing and marital status, were predictive of the total QOL scores in the four domains. This calls for urgent societal and political efforts to strengthen the social living conditions of Syrian refugees in Germany. Further research is needed to gather evidence on the integration mechanisms and social relationships of the Syrian refugees with local communities to understand the determinants of a better QOL of refugees in Germany. In addition, focusing on the components of the domains used in measuring the score of quality of life as showed a direct association to the asylum duration for the refugee population in the current study, which may extend to broader associations with the socio-demographic characteristics of the refugee population such as education, marital status and age groups.

## Data Availability

The datasets used and analyzed during the current study are available from the corresponding author on reasonable request.

## Abbreviations

WHOQOL-BREF: World Health Organization quality of life Instrument, Short Form
QOL: quality of life
BAMF: the Federal Office for Migration and Asylum in Germany
Q1: How would you rate your quality of life
Q2: How satisfied are you with your health

## References

[1] Internationales Komitee vom Roten Kreuz. Ein Jahrzehnt an Verlusten: Syriens Jugend nach zehn Jahren Kreise; 02.2021.

[2] Gulacti U, Lok U, Polat H (2017). Emergency department visits of Syrian refugees and the cost of their healthcare. Pathog Glob Health.

[3] Ibrahim H, Hassan CQ (2017). Post-traumatic stress disorder symptoms resulting from torture and other traumatic events among Syrian Kurdish refugees in Kurdistan region. Iraq Front Psychol.

[4] Johnson H, Thompson A (2008). The development and maintenance of post-traumatic stress disorder (PTSD) in civilian adult survivors of war trauma and torture: a review. Clin Psychol Rev.

[5] Qouta S, Punamäki R-L, El Sarraj E (2003). Prevalence and determinants of PTSD among Palestinian children exposed to military violence. Eur Child Adolesc Psychiatry.

[6] Abdo N, Sweidan F, Batieha A (2019). Quality-of-life among Syrian refugees residing outside camps in Jordan relative to Jordanians and other countries. PeerJ..

[7] Aziz IA, Hutchinson CV, Maltby J (2014). Quality of life of Syrian refugees living in camps in the Kurdistan region of Iraq. PeerJ..

[8] Eckstein B (2011). Primary care for refugees. Am Fam Physician.

[9] Gerritsen AAM, Bramsen I, Devillé W, van Willigen LHM, Hovens JE, van der Ploeg HM (2006). Physical and mental health of afghan, Iranian and Somali asylum seekers and refugees living in the Netherlands. Soc Psychiatry Psychiatr Epidemiol.

[10] Wagner J, Burke G, Kuoch T, Scully M, Armeli S, Rajan TV (2013). Trauma, healthcare access, and health outcomes among southeast Asian refugees in Connecticut. J Immigr Minor Health.

[11] Wong EC, Marshall GN, Schell TL, Elliott MN, Hambarsoomians K, Chun C-A, Berthold SM (2006). Barriers to mental health care utilization for U.S. Cambodian refugees. J Consult Clin Psychol.

[12] Kim G, Worley CB, Allen RS, Vinson L, Crowther MR, Parmelee P, Chiriboga DA (2011). Vulnerability of older Latino and Asian immigrants with limited English proficiency. J Am Geriatr Soc.

[13] Carswell K, Blackburn P, Barker C (2011). The relationship between trauma, post-migration problems and the psychological well-being of refugees and asylum seekers. Int J Soc Psychiatry.

[14] van der Boor CF, Amos R, Nevitt S, Dowrick C, White RG (2020). Systematic review of factors associated with quality of life of asylum seekers and refugees in high-income countries. Confl Health.

[15] Bundesamt für Migration und Flüchtlinge. Aktuelle Zahlen; 2019.

[16] Bundesamt für Migration und Flüchtlinge. Aktuelle Zahlen; 2015.

[17] Bundesamt für Migration und Flüchtlinge. Aktuelle Zahlen; 2016.

[18] Bundesamt für Migration und Flüchtlinge. Aktuelle Zahlen; 2017.

[19] Bundesamt für Migration und Flüchtlinge. Aktuelle Zahlen; 2018.

[20] Statistisches Bundesamt. statistisches jahrbuch: Deutschland und Internationales; 2020.

[21] Rich A-K. Asylerstantragsteller in Deutschland im Jahr 2015: Sozialstruktur, Qualifikationsniveau undBerufstätigkeit.: (BAMF-Kurzanalyse, 3–2016). Nürnberg: Bundesamt für Migration und Flüchtlinge (BAMF) Forschungszentrum Migration, Integration und Asyl (FZ). 2; 2016.

[22] Ragab NJ, Rahmeier L, Siegel M, editors. Mapping the Syrian diaspora in Germany: Contributions to peace, reconstruction and potentials for collaboration with German Development Cooperation; 2017.

[23] Lee RM (2005). Resilience against discrimination: ethnic identity and other-group orientation as protective factors for Korean Americans. J Couns Psychol.

[24] Correa-Velez I, Gifford SM, Barnett AG (2010). Longing to belong: social inclusion and wellbeing among youth with refugee backgrounds in the first three years in Melbourne. Australia Soc Sci Med.

[25] Mossakowski KN (2003). Coping with perceived discrimination: does ethnic identity protect mental health?. J Health Soc Behav.

[26] Cooper RN, Layard R (2005). Happiness: lessons from a new science. Foreign Affairs.

[27] OECD. HOW'S LIFE? 2020 [Place of publication not identified]: ORGANIZATION FOR ECONOMIC; 2020.

[28] OECD (2020). Statistics working papers.

[29] Vahedi S (2010). World Health Organization quality-of-life scale (WHOQOL-BREF): analyses of their item response theory properties based on the graded responses model. Iran J Psychiatry.

[30] Malibary H, Zagzoog MM, Banjari MA, Bamashmous RO, Omer AR (2019). Quality of life (QoL) among medical students in Saudi Arabia: a study using the WHOQOL-BREF instrument. BMC Med Educ.

[31] Group W (1994). Development of the WHOQOL: rationale and current status. Int J Ment Health.

[32] Silva PAB, Soares SM, Santos JFG, Silva LB (2014). Cut-off point for WHOQOL-bref as a measure of quality of life of older adults. Rev Saude Publica.

[33] Hawthorne G, Herrman H, Murphy B (2006). Interpreting the WHOQOL-Brèf: preliminary population norms and effect sizes. Soc Indic Res.

[34] Adedeji A, Bullinger M (2019). Subjective integration and quality of life of sub-Saharan African migrants in Germany. Public Health.

[35] Group TW (1998). The World Health Organization quality of life assessment (WHOQOL): development and general psychometric properties. Soc Sci Med.

[36] Development of the World Health Organization WHOQOL-BREF quality of life assessment (1998). The WHOQOL group. Psychol Med.

[37] Skevington SM, Lotfy M, O'Connell KA (2004). The World Health Organization's WHOQOL-BREF quality of life assessment: psychometric properties and results of the international field trial. A report from the WHOQOL group. Qual Life Res.

[38] Nedjat S, Montazeri A, Holakouie K, Mohammad K, Majdzadeh R (2008). Psychometric properties of the Iranian interview-administered version of the World Health Organization's quality of life questionnaire (WHOQOL-BREF): a population-based study. BMC Health Serv Res.

[39] Gholami A, Araghi MT, Shamsabadi F, Bayat M, Dabirkhani F, Moradpour F, Mansori K, Moradi Y, Rajabi A (2016). Application of the World Health Organization quality of life instrument, short form (WHOQOL-BREF) to patients with cataract. Epidemiol Health.

[40] Gholami A, Jahromi LM, Zarei E, Dehghan A (2013). Application of WHOQOL-BREF in measuring quality of life in health-care staff. Int J Prev Med.

[41] Asnani MR, Lipps GE, Reid ME (2009). Utility of WHOQOL-BREF in measuring quality of life in sickle cell disease. Health Qual Life Outcomes.

[42] Mazaheri M (2010). Overall, and specific life satisfaction domains: preliminary Iranian students norms. Iran J Public Health.

[43] World health organization. WHOQOL-BREF, Introduction,administration, scoring,and generic version of the assessment. 1996.

[44] Statistisches Bundesamt. statistisches jahrbuch: Deutschland und Internationales; 2019.

[45] Fozdar F, Torezani S (2008). Discrimination and well-being: perceptions of refugees in Western Australia. Int Migr Rev.

[46] Sevillano V, Basabe N, Bobowik M, Aierdi X (2014). Health-related quality of life, ethnicity and perceived discrimination among immigrants and natives in Spain. Ethn Health.

[47] Cummins RA (2000). Objective and subjective quality of life: an interactive model. Soc Indic Res.

[48] Lawton MP, Winter L, Kleban MH, Ruckdeschel K (1999). Affect and quality of life: objective and subjective. J Aging Health.

[49] Gottvall M, Sjölund S, Arwidson C, Saboonchi F (2019). Health-related quality of life among Syrian refugees resettled in Sweden. Qual Life Res.

[50] de Vries J, van Heck GL (1994). Quality of life and refugees. Int J Ment Health.

[51] Laban CJ, Komproe IH, Gernaat HBPE, de Jong JTVM (2008). The impact of a long asylum procedure on quality of life, disability and physical health in Iraqi asylum seekers in the Netherlands. Soc Psychiatry Psychiatr Epidemiol.

[52] Haj-Younes J, Strømme EM, Igland J, Kumar B, Abildsnes E, Hasha W, Diaz E (2020). Changes in self-rated health and quality of life among Syrian refugees migrating to Norway: a prospective longitudinal study. Int J Equity Health.

[53] Löfvander M, Rosenblad A, Wiklund T, Bennström H, Leppert J (2014). A case-control study of self-reported health, quality-of-life and general functioning among recent immigrants and age- and sex-matched Swedish-born controls. Scand J Public Health.

[54] Mehdizadeh Kashi A, Moradi Y, Chaichian S, Najmi Z, Mansori K, Salehin F, Rastgar A, Khateri S (2018). Application of the World Health Organization quality of life instrument, short form (WHOQOL-BREF) to patients with endometriosis. Obstet Gynecol Sci.

[55] Grochtdreis T, König H-H, Riedel-Heller SG, Dams J. Health-related quality of life of asylum seekers and refugees in Germany: a cross-sectional study with data from the German socio-economic panel. Applied Research Quality Life. 2020. 10.1007/s11482-020-09877-4.

[56] Uygun E (2020). The relation between Syrians' quality of life, depression and anxiety levels and economic conditions: a cross-sectional study at an adult refugee mental health clinic in Turkey. Anadolu Psikiyatri Derg.

[57] Carlsson JM, Mortensen EL, Kastrup M (2006). Predictors of mental health and quality of life in male tortured refugees. Nord J Psychiatry.

[58] Carlsson JM, Olsen DR, Mortensen EL, Kastrup M (2006). Mental health and health-related quality of life: a 10-year follow-up of tortured refugees. J Nerv Ment Dis.

[59] Gerritsen AAM, Devillé W, van der Linden FAH, Bramsen I, van Willigen LHM, Hovens JEJM, van der Ploeg HM (2006). Psychische en lichamelijke gezondheidsproblemen van en gebruik van zorg door Afghaanse, Iraanse en Somalische asielzoekers en vluchtelingen [mental and physical health problems of, and the use of healthcare by, afghan, Iranian and Somali asylum seekers and refugees]. Ned Tijdschr Geneeskd.

[60] Redko C, Rogers N, Bule L, Siad H, Choh A (2015). Development and validation of the Somali WHOQOL-BREF among refugees living in the USA. Qual Life Res.

[61] Daher AM, Ibrahim HS, Daher TM, Anbori AK (2011). Health related quality of life among Iraqi immigrants settled in Malaysia. BMC Public Health.

[62] von Werthern M, Robjant K, Chui Z, Schon R, Ottisova L, Mason C, Katona C (2018). The impact of immigration detention on mental health: a systematic review. BMC Psychiatry.

[63] Leiler A, Bjärtå A, Ekdahl J, Wasteson E (2019). Mental health and quality of life among asylum seekers and refugees living in refugee housing facilities in Sweden. Soc Psychiatry Psychiatr Epidemiol.

[64] Mikolajczyk RT, Maxwell AE, Eljedi A, Preedy VR, Watson RR (2010). Quality of life and chronic illness among refugee populations. Handbook of disease burdens and quality of life measures: with 1001 tables.

[65] Georgiadou E, Schmitt GM, Erim Y (2020). Does the separation from marital partners of Syrian refugees with a residence permit in Germany have an impact on their quality of life?. J Psychosom Res.

[66] Miller A, Hess JM, Bybee D, Goodkind JR (2018). Understanding the mental health consequences of family separation for refugees: implications for policy and practice. Am J Orthop.

[67] Correa-Velez I, Green A, Murray K, Schweitzer RD, Vromans L, Lenette C, Brough M (2020). Social context matters: predictors of quality of life among recently arrived refugee women-at-risk living in Australia. J Immigr Refug Stud.

[68] Crea TM, Calvo R, Loughry M (2015). Refugee health and wellbeing: differences between urban and camp-based environments in sub-Saharan Africa. J Refug Stud.

[69] Rueda S, Raboud J, Mustard C, Bayoumi A, Lavis JN, Rourke SB (2011). Employment status is associated with both physical and mental health quality of life in people living with HIV. AIDS Care.

[70] von dem Knesebeck O, Geyer S (2007). Emotional support, education and self-rated health in 22 European countries. BMC Public Health.

[71] Warr P, Butcher V, Robertson I, Callinan M (2004). Older people's well-being as a function of employment, retirement, environmental characteristics and role preference. Br J Psychol.

[72] Higgins CA, Duxbury LE (1992). Work—family conflict: a comparison of dual-career and traditional-career men. J Organiz Behav.

[73] Young MY (2001). Moderators of stress in Salvadoran refugees: the role of social and personal resources. Int Migr Rev.

[74] Wiley Online Library. Moderators of Stress in Salvadoran Refugees: The Role of Social and Personal Resources1. 18.01.2021. Accessed 18 Jan 2021.

[75] Lerdal A, Andenæs R, Bjørnsborg E, Bonsaksen T, Borge L, Christiansen B, Eide H, Hvinden K, Fagermoen MS (2011). Personal factors associated with health-related quality of life in persons with morbid obesity on treatment waiting lists in Norway. Qual Life Res.

